# Time-Specific Ecologic Niche Models Forecast the Risk of Hemorrhagic Fever with Renal Syndrome in Dongting Lake District, China, 2005–2010

**DOI:** 10.1371/journal.pone.0106839

**Published:** 2014-09-03

**Authors:** Hai-Ning Liu, Li-Dong Gao, Gerardo Chowell, Shi-Xiong Hu, Xiao-Ling Lin, Xiu-Jun Li, Gui-Hua Ma, Ru Huang, Hui-Suo Yang, Huaiyu Tian, Hong Xiao

**Affiliations:** 1 College of Resources and Environment Science, Hunan Normal University, Changsha, China; 2 Hunan Provincial Center for Disease Control and Prevention, Changsha, China; 3 Division of International Epidemiology and Population Studies, Fogarty International Center, National Institutes of Health, Bethesda, Maryland, United States of America; 4 Simon A. Levin Mathematical, Computational & Modeling Sciences Center, School of Human Evolution and Social Change, Arizona State University, Tempe, Arizona, United States of America; 5 School of Public Health, Shandong University, Jinan, China; 6 Center for Disease Control and Prevention of Beijing Military Region, Beijing, China; The Scripps Research Institute, United States of America

## Abstract

**Background:**

Hemorrhagic fever with renal syndrome (HFRS), a rodent-borne infectious disease, is one of the most serious public health threats in China. Increasing our understanding of the spatial and temporal patterns of HFRS infections could guide local prevention and control strategies.

**Methodology/Principal Findings:**

We employed statistical models to analyze HFRS case data together with environmental data from the Dongting Lake district during 2005–2010. Specifically, time-specific ecologic niche models (ENMs) were used to quantify and identify risk factors associated with HFRS transmission as well as forecast seasonal variation in risk across geographic areas. Results showed that the Maximum Entropy model provided the best predictive ability (AUC = 0.755). Time-specific Maximum Entropy models showed that the potential risk areas of HFRS significantly varied across seasons. High-risk areas were mainly found in the southeastern and southwestern areas of the Dongting Lake district. Our findings based on models focused on the spring and winter seasons showed particularly good performance. The potential risk areas were smaller in March, May and August compared with those identified for June, July and October to December. Both normalized difference vegetation index (NDVI) and land use types were found to be the dominant risk factors.

**Conclusions/Significance:**

Our findings indicate that time-specific ENMs provide a useful tool to forecast the spatial and temporal risk of HFRS.

## Introduction

Hemorrhagic fever with renal syndrome (HFRS), a rodent borne disease caused by hantaviruses, is clinically characterized by fever, haemorrhage, headache, back pain, abdominal pain and acute kidney damage in humans [1]. China currently has the highest incidence of HFRS globally; approximately 90% of the total HFRS case incidence is reported in this country [2]. In particular, Hunan Province in south-central China reports one of the highest HFRS incidence rates in China. The Dongting Lake district, which is located in northeastern part of the Hunan province and is known as “the land of fish and rice”, is considered a typical hotspot of HFRS [3]. The number of reported cases in Dongting Lake district has been as high as 2,232 HFRS cases in 1995.

The transmission of HFRS has been closely associated with rodent populations, land use patterns, elevation, vegetation types [2], , as well as temperature, humidity, and rainfall [1]. Rodent habitat and rodent behavior can be influenced by temperature, precipitation and land use [6], [7], thus indirectly affecting the transmission dynamics of HFRS. Based on ecological niche models (ENMs), Wei et al. found that risk areas of hantaviruses infections in rodents coincided with human HFRS cases, and the distribution of infected rodents was closely correlated with land cover and elevation in the Shandong Province [8]. We have previously employed maximum entropy models to successfully predict the potential risk areas of HFRS in the middle and lower reaches of Xiangjiang River in China [9]. Furthermore, we have also found that the HFRS transmission in Changsha is significantly influenced by elevation, temperature and rainfall by using genetic algorithms in a rule-set production (GARP) model [10]. ENMs have also been used to explore the ecological requirements of disease and predict its potential distribution [11], [12]. However, there is a scarcity of studies that link temporal variations in disease incidence with the corresponding environment. Time-specific ENMs have been proved useful to predict the transmission risk of disease in both space and time and characterize the dynamic ecological requirements [13].

The ecological niche comprised a set of environmental conditions that allow a species to maintain its population over time. Hence, the ecological niche of a species may be conservative and could remain unchanged for a long time. ENMs can be used to exploit the conservatism of ecological niches to explore the ecological demands of a species [14]. By combining data on human cases, hosts, pathogens and their environment, ENMs can be useful to quantitatively relate the occurrence of diseases with environmental variations, analyze transmission patterns at different spatial-temporal scales, identify risk factors, and predict potential risk areas. Hence, ENMs provide a useful tool to support public health decision making for prevention and control of diseases [15].

In this study, we evaluated GARP, Maximum Entropy models, logistic regression models and DOMAIN model together with human HFRS records and environmental variables to generate predictions of HFRS risk in the Dongting Lake district, China. Time-series ENMs based on the optimal model were also constructed to investigate the seasonal variations of risk across areas in the Dongting Lake district.

## Materials and Methods

### Study area

**Figure 1:**
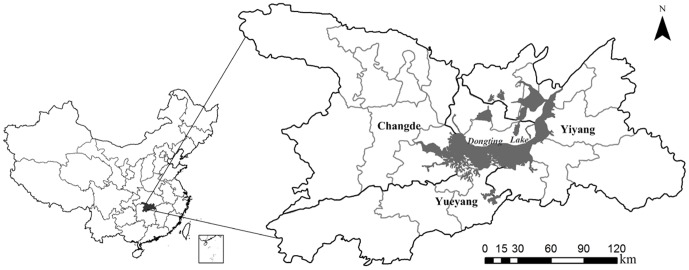
Location of study area, showing Dongting Lake district, China, 2005–2010.

Dongting Lake is located in the northeast of Hunan Province in China, between 28°30′∼30°20′ N and 110°40′∼113°10′ E. It is the second largest fresh water lake in China, covering 2,820 km^2^. The Dongting Lake district has a subtropical humid monsoon climate with annual average temperature of 16.4–17.0°C, an annual average precipitation ranging 1200–1550 mm, and an annual average humidity of 80%. Hence, this setting provides a potentially suitable environment for rodent populations and HFRS transmission. Hunan Province is a well-established HFRS epidemic area where the Dongting Lake district is one of the most affected epidemic areas in this province [3], [16]. The Dongting Lake district comprising Yueyang, Changde and Yiyang around the Dongting Lake were selected for our study region (Figure 1).

### Data collection and management

Data on HFRS cases reported in Yueyang, Changde and Yiyang city from 2005 to 2010 were obtained from the Hunan Center for Disease Control and Prevention (CDC). A total of 296 HFRS cases during the 6-year period were initially diagnosed clinically according to diagnostic criteria from the Ministry of Health of the People's Republic of China [17]. Each case record contained information about sex, age, residential address, and the date of onset of symptoms, but it did not include information to distinguish infections caused by different types of hantaviruses. All cases that were geocoded by residential address using Google Earth were distributed mainly in the southeast and southwest of the district, near Dongting lake.

Data on precipitation and temperature from 2005 to 2009 were obtained from the China Meteorological Data Sharing Service System. Land use type data were obtained from the Second National Land Survey and were categorized as cultivated land, forest, grass, residential land, water, permanent wetlands and barren or sparsely vegetated by remote sensing images and an uniform standard of visual interpretation. If land use types or classification boundary can not be distinguished clearly in remote sensing images, then field survey and measurements on the spot are needed to define land use types. The compound topographic index (CTI) was obtained from the United States Geological Survey. CTI is also known as the topographic wetness index, which is calculated by the confluence area of upstream and the landscape slope. CTI codes a comprehensive terrain variable, which water and sediment transport in a particular landscape, is a very effective metric to predict soil attributes. Elevation values were derived from the digital elevation model (DEM) with a spatial resolution of 1 km. Slope was calculated from DEM data. The Human Footprint Index (Geographic), a comprehensive reflection of population density, infrastructure, land use, and dredge shipping and road construction, was obtained from the Center for International Earth Science Information. The Monthly normalized difference vegetation index (NDVI) value was obtained from the International Scientific Data Service Platform. Data on eco-geographical characteristics were obtained from the International Environmental Protection Organization Association, and the distance to the nearest water source was calculated from maps (Table 1). Data were analyzed in ArcGIS 9.3 (ESRI Inc., Redlands, CA, USA), and the environmental variables were resampled to generate a raster dataset with a spatial resolution of 0.00833° (nearly 1 km).

**Table 1:** List of environmental variabl used in ENMs to assess HFRS potential risk levels in Dongting Lake District, 2005 to 2009.

Variables	Source	Type and time
**HFRS cases**	Hunan Center for Disease Control and Prevention	Monthly data, 2005–2010.
**DEM**	Geospatial Data Cloud (http://datamirror.csdb.cn)	2005–2009
**Temperature**	China Meteorological Data Sharing Service System (http://cdc.cma.gov.cn)	Average annual data, 2005–2009.
**Precipitation**	China Meteorological Data Sharing Service System (http://cdc.cma.gov.cn)	Average annual data, 2005–2009.
**NDVI**	International Scientific Data Service Platform (http://datamirror.csdb.cn/)	Average monthly data, 2005–2009.
**Eco-geographical data**	International Environmental Protection Organization Association (http://iepoasc.cn.china.cn)	Average annual data, 2005–2009.
**Land use types**	the Second National Land Survey	Yearly data, 2005.
**Human Footprint Index (Geographic)**	Center for International Earth Science Information (http://www.ciesin.columbia.edu/)	Average annual data, 1995–2004.
**CTI**	United States Geological Survey (http://eros.usgs.gov)	Average annual data
**Distance to water source**	Calculated in ArcGIS 9.3	Average annual data, 2005–2009.
**Slope**	Calculated in ArcGIS 9.3	Average annual data, 2005–2009.

### Ecological niche models

GARP, an integrated spatial analysis system for predicting the distribution of species, is composed of a set of rules, or if-then relationships [15]. The set of rules is developed through evolutionary refinement by testing and selecting rules on random subsets of training data sets to explore the relationship between the non-random distribution of species and the environment [15], [18]. The primary principle of the GARP model is to use iterative calculation, selection rules, evaluation, verification, include or reject so that the final outcome is an optimal model [19]. Occurrence points were divided randomly into two parts: an extrinsic testing dataset (50%), which was used for model evaluation, and a training dataset (50%) which was used for model development. The average area under the receiver operating characteristic (ROC) curve (AUC) was used to evaluate predictive accuracy of the GARP model. Here HFRS cases and environmental variables from 2005 to 2009 were iteratively calculated to obtain 10 models. Then an overall average model was obtained by overlapping the resulting 10 models and was tested by using the remainder HFRS cases reported in 2010. Finally, HFRS cases in 2010 were assigned the value of 1, while 1,000 randomly sampled points in the study region were assigned the value of 0 to calculate predictive accuracy.

MaxEnt is a machine learning method, which estimates density and species distributions by finding the probability distribution of maximum entropy to constraints representing an unknown distribution [20], [21]. Based on the known distribution of HFRS cases and environmental data, MaxEnt simulated niche requirements for the species and inferred the potential distribution [20], [21]. Maximum entropy probability distributions were obtained from HFRS data and environmental variables iterated 10 times in this study. Then the maximum probability occurrence model was calibrated based on the background data.

Logistic regression modeling is a spatial modeling method of biodiversity for determining potential habitat. Logistic regression is a probabilistic non-linear regression, and it is a common statistical analysis method that defines dependent variables as the qualitative variables [22]. The HFRS cases occurrence rate is calculated by the index of the parameter estimate value to export independent variables, which affect the probability of HFRS in logistic regression. Multivariable logistic regression methods based on data sampling calculate regression coefficients (occurrence rate) for each independent variable. The corresponding regression coefficient is interpreted as the rate of change of the logit function per unit of change of a specific variable. Here, cases from 2005 to 2009 were assigned the value of 1, and 1,000 randomly points were assigned the value of 0. Analyses were carried out using a backward stepwise procedure in the stats library of R.

The DOMAIN model uses the Gower metric to calculate a point-to-point similarity based on distance and the proximity in the environmental space; the point of the maximum similarity is selected by comparing the point-to-point similarity within a certain distance [23]. The Gower metric can provide a suitable means of quantifying similarity between two points, which uses range standardization to equalize the contribution from each environmental attribute. This method of standardization is preferred over variance standardization in this application because it is less prone to bias arising from dense clusters of the sampled points. Similarity is calculated as 1 minus the standard distance between two points. The maximum similarity values generated are not probability estimates, but degrees of classification confidence. DOMAIN is based on a continuous similar function, which is flexible for the simulation of the species distribution [23].

Time-specific ENMs were constructed to predict disease dynamic transmission changes in time and space based on the optimal ENM that was previously calculated. Monthly HFRS occurrences, environmental data and monthly NDVI, as well as differences between the particular month and the two previous months were included in the modeling process. A total of three month-specific remotely sensed data layers were selected on different time scales to capture changes in the environment across seasons.

## Results and Analysis

### Comparisons of ENMs results

**Figure 2:**
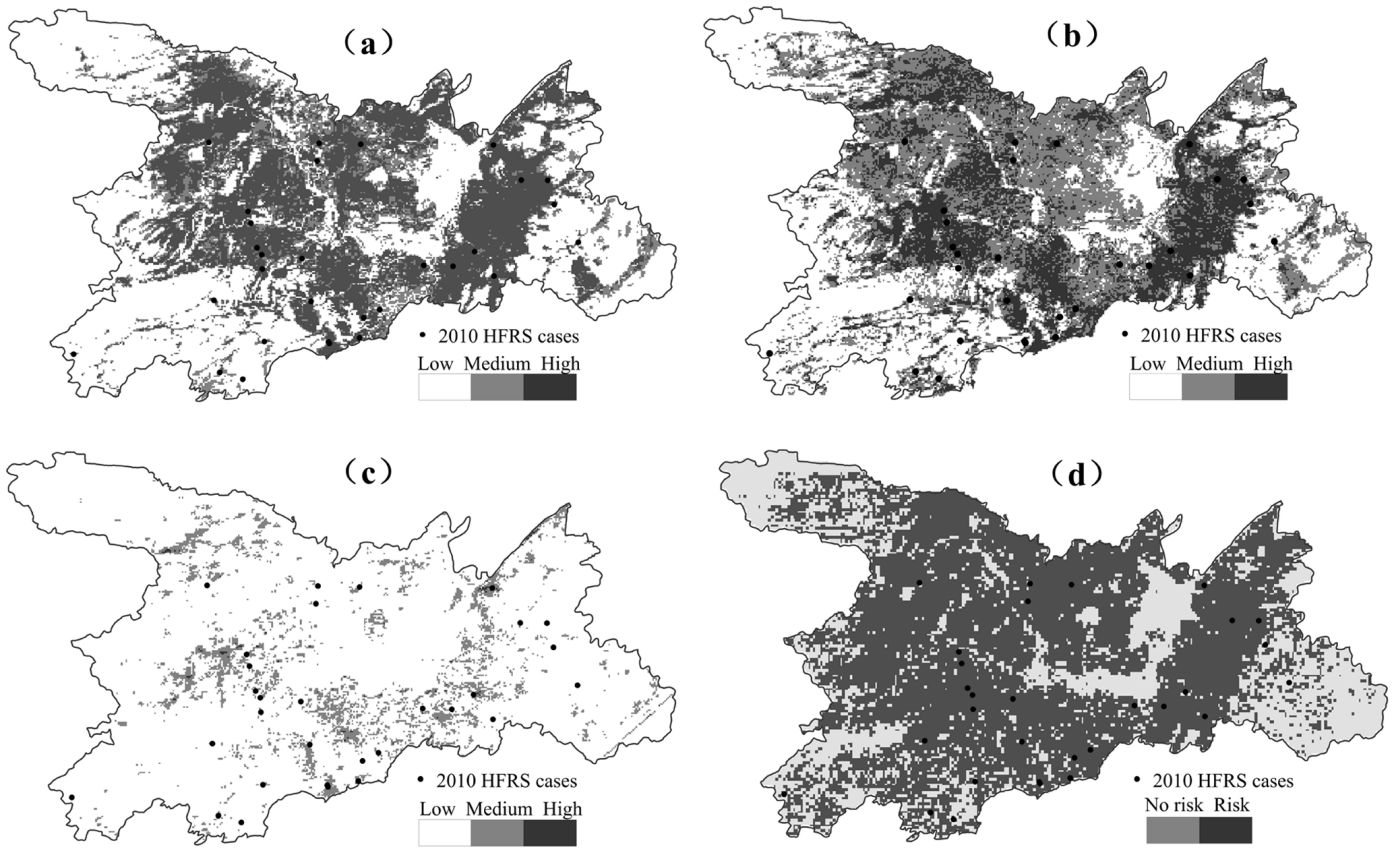
Results of ENMs predictions of HFRS potential risk areas in Dongting Lake district, 2010. (a) predictions by GARP model; (b) by MaxEnt model; (c) by Logistic model; (d) by Domain model.

Thirty eight HFRS cases, distributed near the southeast and southwest of Dongting lake in 2010, were used to validate the predictive power of our model. The predicted risk areas of the GARP model were categorized into low-risk (<0.3), medium-risk (0.3 to 0.6), and high-risk areas (0.6 to 0.9). Findings indicated that only 4 out of 38 cases were distributed at low-risk areas while the remainder of the cases occurred at high-risk areas (Figure 2a). Evaluation data were merged with 10,000 randomly selected background points and entered into a ROC analysis together with all of the cases that occurred in 2010 in order to derive an AUC estimate, a measure of the predictive accuracy of the GARP model. The AUC was estimated at 0.723 (95% CI: 0.671∼0.774, SD = 0.026, *P*<0.001).

The results of the MaxEnt model showed that the monthly average NDVI (from April to September and November; cumulative contribution rate of 62.7%), land use (cumulative contribution rate of 7.9%), human footprint index (contribution rate of 7.8%), DEM (contribution rate of 5.5%), ecosystem (contribution rate of 4.5%), distance from the water (contribution rate of 3.1%) and CTI (contribution rate of 2.7%) were the main environmental variables that influenced the spread of HFRS. Our results showed that predicted high-risk areas were consistent with the actual distribution of cases in 2010; 8 out of 38 cases were distributed at medium risk areas while the remainder cases occurred in high-risk areas (Figure 2b). The corresponding AUC was estimated at 0.775 (SD = 0.04).

Predictions derived from the logistic model indicated that the occurrence of HFRS was associated with NDVI in the months of May, June and November as well as with CTI, DEM, the human footprint index, land use, and temperature. The predicted risk areas were divided into low-risk areas (<0.3), away from the Dongting Lake area, medium risk areas (0.3 to 0.6), and high-risk areas (0.6 to 0.9) which were distributed in the southeast and west of the study area. Verification results with 38 cases of HFRS showed most cases were in the low-risk areas with a corresponding AUC estimated at 0.746 (95% CI: 0.715∼0.777, SD = 0.016, *P*<0.001) (Figure 2c).

NDVI covering the months April-June, October and November, CTI, DEM, and distance from water source turned out to be the main environmental variables influencing the transmission of HFRS in the Dongting Lake area based on the DOMAIN model. The predicted risk areas were mainly distributed around Dongting Lake (Figure 2d) while the AUC was estimated at 0.651.

A comparison of the four models indicated that low-risk areas of HFRS incidence always occurred far away from Dongting Lake whereas the high risk areas always focused on the southeast and northwest of Dongting Lake. These results were consistent with the actual HFRS case distribution in 2010. The predicted HFRS risk zones were similar according to the GARP and MaxEnt models, except for areas in the northwest of the Dongting Lake which were classified as high-risk according to the GARP model. While these areas resulted in medium risk based on the MaxEnt model, the results of MaxEnt were more consistent with the actual case distribution in 2010. A comparison of the results obtained from the MaxEnt and logistic models revealed that high and medium risk areas yielded by the logistic model were smaller than those predicted by the MaxEnt model. The validation cases were mostly located in low-risk areas. Results from the DOMAIN model only demonstrated two disease regions indicating occurrence or non-occurrence. However, this analysis did not yield a clear prediction of risk as the accuracy of this prediction was not high. Based on the prediction accuracy, the MaxEnt model had the largest AUC value (0.775) followed by the logistic model. The DOMAIN had the smallest AUC value (0.651). Hence, the MaxEnt model provided the highest predictive power of all studied models.

### The results of time-specific ENMs

**Figure 3:**
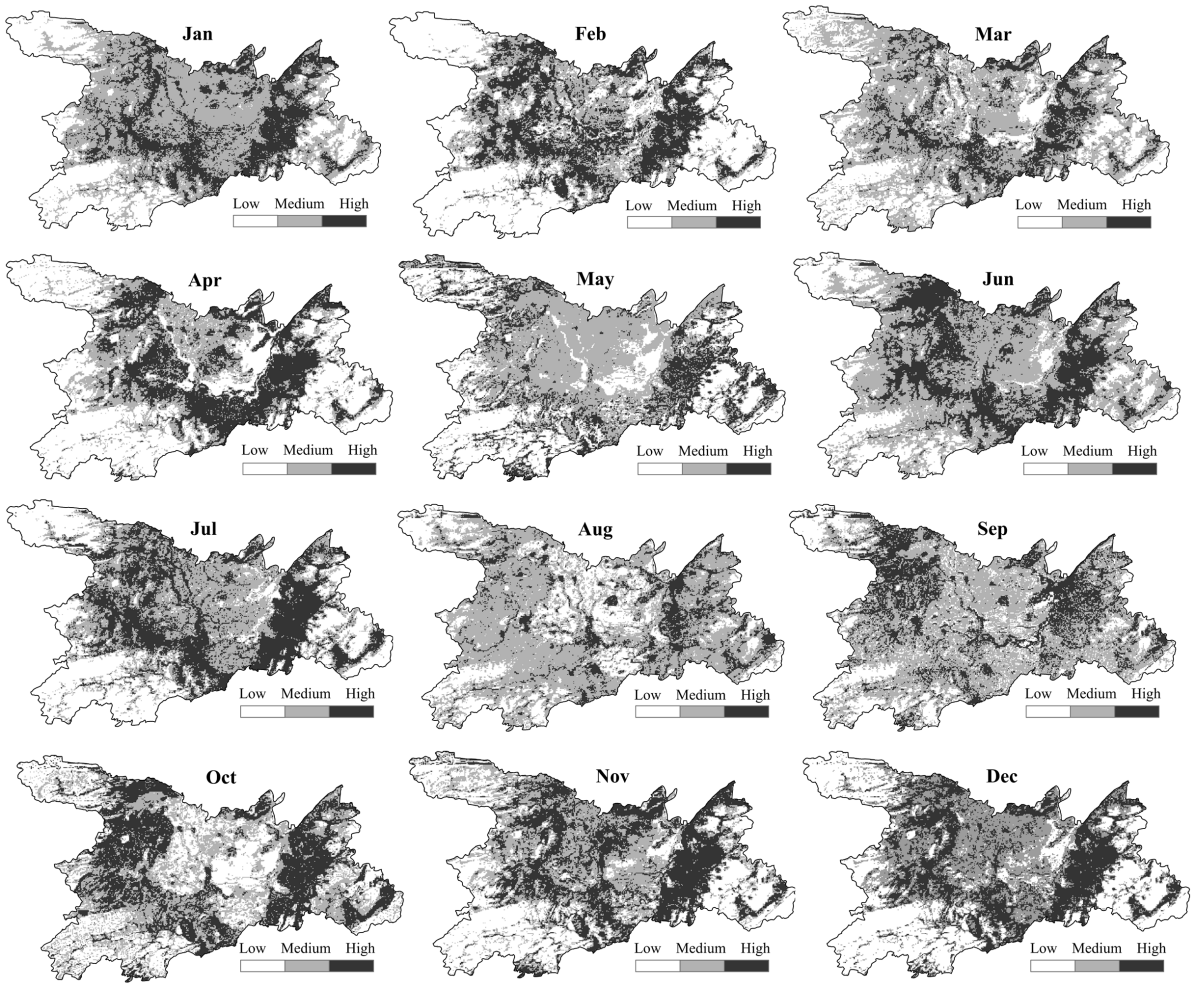
The results of time-specific ENMs highlighting temporal changes in HFRS potential risk levels in Dongting Lake district.

Compared with ENMs, results obtained from the time-specific ENMs were more precise and rigorous. Not surprisingly our modeling results showed that risk areas changed according to seasons. Specifically, the area of HFRS risk was limited in March, May and August while high-risk areas occurred in June, July and October to December and were mainly distributed around Dongting Lake and showed statistically significantly higher risk than in other months. High-risk areas always appeared southeast of the Dongting Lake while low-risk areas always clustered southwest and northwest of our study area (Figure 3). The models provided the best performance during spring and winter. Over 50% of monthly cases were clustered in high-risk areas (Table 2).

**Table 2:** The results of time-specific ENMs predictions of HFRS potential risk areas in Dongting Lake district, 2010.

time-specific ENMs	Low risk area		Moderate risk area		High risk area		AUC
	incidence	area (%)	incidence	area (%)	incidence	area (%)	
Jan	0	25.4%	0	48.9%	100%	25.5%	0.748
Feb	0	40.5%	33.3%	30.7%	66.7%	28.6%	0.766
Mar	0	28.5%	100%	52.6%	0	18.9%	0.697
Apr	20%	42.8%	20%	29.4%	60%	27.8%	0.762
May	0	32.4%	0	47.7%	0	19.9%	0.733
Jun	0	20.2%	0	50.6%	100%	29.2%	0.775
Jul	0	30.2%	50%	36.8%	50%	33.0%	0.652
Aug	0	25.6%	0	58.6%	100%	15.7%	0.670
Sep	0	20.4%	0	54.2%	100%	25.5%	0.683
Oct	0	31.0%	0	36.0%	0	33.1%	0.661
Nov	0	32.2%	14.3%	34.6%	85.7%	33.3%	0.675
Dec	0	35.5%	18.2%	35.0%	81.8%	29.5%	0.713

Our findings obtained from the Jackknife method showed that NDVI was the main factor associated with the HFRS case distribution followed by land use, DEM, and the landscape slope. Differences in NDVI between a particular month and the two previous months did not have much influence on HFRS. NDVI in November, December and January had little impact on HFRS about two months later. NDVI in April, May, November and December were found to be the main risk factors for HFRS. The highest incidence of HFRS was in spring (NDVI was between 0.5 and 0.7) and in winter (NDVI was between 0.4 and 0.5). DEM had a great impact on HFRS transmission in January, February, June and July, and the risk of HFRS decreased with the increase of DEM. Moreover, construction land was the main risk land type associated with HFRS transmission between June and September (Table 3).

**Table 3:** Changes in contributions of variables obtained from time-specific ENMs (%) predictions of HFRS potential risk areas in Dongting Lake district, 2010.

	NDVI	NDVI (t-1)	NDVI (t-2)	Land use	DEM	Slope
Jan	7.9	1.8	8.2	18.1	45.1	19
Feb	24.3	1.8	8.5	10.8	50.8	3.7
Mar	27.9	1.5	10.4	7	16.3	36.9
Apr	52.4	0.1	0.1	22.5	2.7	22.2
May	54.5	2.9	0.9	33.3	1	7.4
Jun	13.7	0	3.6	45.1	36.6	1
Jul	20.3	0.9	2.2	48.8	19.9	8.3
Aug	47.4	1	4.2	28.6	8.2	10.7
Sep	42.4	5	1.6	31.5	9.8	9.7
Oct	41.9	1.4	0.2	6.8	2.6	47.1
Nov	73.9	6	1.4	4.7	1.5	12.5
Dec	54.8	4.9	3.4	17.5	3.9	15.4

## Discussion

We have explored the environmental risk factors and forecasted potential risk areas for HFRS infections in the Dongting Lake district by using time-specific ENMs. Using the best model according to AUC values, we generated time series ENMs for further assessment of potential risk areas, and investigated the temporal and spatial distribution patterns of HFRS in the Dongting Lake district. High-risk areas predicted by the MaxEnt model were consistent with HFRS cases reported in 2010, providing the maximum AUC value among all the four models. Time-specific ENMs showed that HFRS incidence was high in the Dongting Lake district, and risk areas varied significantly across seasons. Model performance was especially good in spring and winter. Predicted risk areas were small in March, May and August, and high in June, July and October to December. High-risk areas were concentrated southeast and southwest of Dongting Lake. NDVI, land use types, DEM and slope were found to be the main risk factors of HFRS transmission.

The higher performance of the models in spring and winter mainly resulted from the high incidence of HFRS, as it peaked in spring and winter. Environmental requirements of disease outbreaks can be better quantified with a larger number of human cases, thus resulting in better predictions for spring and winter. Seasonal variations in risk areas indicated that HFRS incidence was closely associated with environmental factors which can affect rodent activities in Dongting Lake district [6], [24]. Dense rodent populations lead to increase contacts between rodents and humans, which directly influence the incidence of HFRS [25], [26]. The incidence of HFRS remained high in the southeast of the Dongting Lake (Yueyang City), which may have resulted from its characteristic high humidity (average humidity of 74%), moderate temperature (annual average temperature of about 18 °C) and geographical location. HFRS epidemic areas were mostly distributed in low-lying wet areas or sub-humid regions [2]. High humidity may not only affect living conditions of host animals, enhance the infectivity and vitality of hantavirus, but also affect vegetation growth, subsequently influencing disease risk [25], [27], [28]. HFRS incidence was closely associated with temperature. Temperature may influence the distribution and activities of rodents. The growth of rodent populations may also be associated with temperature, as temperature may affect the pregnancy rate, litter size, birth rate and survival rate of rodent populations [9], [28], [29].

Our study showed that NDVI, land use type, DEM and landscape slope were the most important risk factors of HFRS transmission in the Dongting Lake district. NDVI reflects the level of vegetation coverage, which comprehensively shows the geographical characteristics [30]. NDVI in persistently highest risk areas had an early onset, with significantly higher levels of green vegetation that lasted longer than at comparable sites [25]. A relatively high NDVI value, with the growing and harvest seasons of rice taking place from April to November, may provide shelters for reproduction and increased activities for rodents [7]. As a possible result of the delay between the reproduction of rodents and outbreaks of diseases among humans, HFRS incidence was the highest in winter when the NDVI value was low (between 0.4 and 0.5). NDVI may also be a good indicator of rodent food availability. For example the fluctuation of food supplies may influence the rodent population density, thus indirectly affecting HFRS incidence[31], [32]. Cultivated land and grassland are the main land use types that affect HFRS incidence. Rodents have a strong habitat selection for highly covered and less disturbed habitats, which are commonly found in agricultural habitats and pastureland habitats [33], [34], [35], [36]. Rodents take advantage of these habitats for rapid reproduction and safe foraging and activity that are not easily captured [34].Vegetation and crops offer cover and food for rodents [36]. The occurrence of HFRS was highest in flat areas, which may be related to land use types. Cultivated lands and residential areas are mainly concentrated on flat areas where contacts between people and rodents are more likely to occur.

Limitations of this study should also been acknowledged. Firstly, the impact of the socio-economic factors and specific human activities on HFRS incidence were not explicitly considered in our study. Secondly, specific species of rodent populations were not characterized in our study area. Finally, HFRS cases were obtained from a passive surveillance system, and hence some cases may have been missed. For example, patients with less serious or non-obvious symptoms may not seek medical care, thus resulting in underreporting of HFRS incidence. However, we focused here on analyzing the spatial-temporal spreading patterns of HFRS rather on estimating the actual burden of HFRS in our study area.

In conclusion, ENMs have so far been mostly employed to explore environmental risk factors and predict the potential risk areas at different spatial scales, without considering temporal variation in risk. In this study, we studied both the temporal and spatial distribution patterns of HFRS in the Dongting Lake district. Our results showed that the potential risk areas were mainly concentrated around Dongting Lake with significant seasonal variation. Our findings support the use of spatial-temporal data to improve our understanding of the transmission patterns of HFRS more accurately and effectively. Our results also provide a quantitative basis to guide local control and prevention measures and have the potential to mitigate the risk and economic loss associated with HFRS. From a public health perspective, our results support the need to carry out deratization campaigns in spring and summer around the Dongting Lake as well as enhance population immunity by vaccination all year long. Overall, disease prevention and control measures need to be strengthened in high-risk areas which vary by month of the year. For instance, public health campaigns aimed to inform the population about the importance of improving ventilation and sanitation of the living conditions in order to reduce the risk of HFRS infection.
